# The Road to TNR: Examining Trap-Neuter-Return Through the Lens of Our Evolving Ethics

**DOI:** 10.3389/fvets.2018.00341

**Published:** 2019-01-11

**Authors:** Peter Joseph Wolf, Joan E. Schaffner

**Affiliations:** ^1^Best Friends Animal Society, Kanab, UT, United States; ^2^The George Washington University Law School, Washington, DC, United States

**Keywords:** cats, feral cats, community cats, trap-neuter-return, TNR, ethics, animal sheltering, public opinion

## Abstract

In the 2008 article “A Review of Feral Cat Control,” Robertson explored the trend developing in the management of so-called “feral” cats away from lethal methods toward the non-lethal method of trap-neuter-return (TNR). The review explored various issues raised by the presence of these unowned, free-roaming cats in our neighborhoods (e.g., zoonotic disease and wildlife predation), stakeholder interests, and management options—all based on then-available information. Missing from the review, however, was an exploration of the shifting ethics underlying TNR's increasing popularity. In this essay, we explore the ethical aspects of community cat management in the U.S. as reflected in the momentum of the “no-kill movement” generally and TNR in particular. We argue that these powerful cultural currents reflect two interrelated ethical theories: (1) a zoocentric ethic that recognizes the intrinsic value of non-human animals beyond any instrumental value to humans, and (2) a virtue ethic that recognizes the legitimacy of “emotional” considerations (e.g., compassion) that rightly accompany decisions about how best to manage community cats.

## Introduction

In “A Review of Feral Cat Control,” Robertson (1) explored the trend developing in the management of unowned, free-roaming (“community”) cats, away from lethal methods toward the non-lethal method of trap-neuter-return (TNR). The review explored various issues raised by the presence of community cats (e.g., zoonotic disease and wildlife predation), stakeholder interests, and management options—all based on then-available information. Seven times Robertson alluded to the ethical implications of allowing these cats in our communities, and of the competing management methods. Missing from the review, however, was an exploration of the shifting ethics underlying TNR's increasing popularity.

In the 10 years since the publication of Robertson's review, TNR has become more widely adopted in communities across the U.S. (2), though the practice remains controversial (3). For these reasons alone, it's worth examining “the rise of TNR” through two different (but presumably related) lenses: ethics and public opinion. Among the questions we're most interested in exploring: What are the ethical underpinnings of TNR, and non-lethal management more generally? And how are these ethics reflected in the public's preference for one management scheme over others?

## Recognizing the Intrinsic Value of Non-Human Animals

As Robertson (1) explained, “the question of ending the life of healthy animals is a far reaching ethical question, as humans do kill healthy animals for food and pest control.” Indeed, the management of community cats was, historically, based almost exclusively in an anthropocentric ethical framework—community cats were trapped and killed (4, 5). Anthropocentric theories assign intrinsic value only to humans, with instrumental value assigned to all other entities based only on their use value (or perceived negative impacts) to humans. Increasingly, however, greater consideration is being given to the intrinsic value of animals—a zoocentric ethic—making their interests morally relevant (6, 7).

This shift from an anthropocentric ethic to a zoocentric ethic is, in part, the result of a growing body of research demonstrating cognition, emotion, and sentience in animals once assumed to be “unfeeling” and relegated to the lower rungs of the now-obsolete “evolutionary ladder” (8, 9). Sentience has become “a criterion of moral significance, of being the kind of entity toward which a moral agent can have moral obligations” (10). As a result, although causing harm to a morally relevant animal is not automatically considered “wrong” in an absolute sense, the moral obligations associated with the recognition of an animal's intrinsic worth require that “the burden of proof is on one wishing to harm or exploit. The contrast is as sharp as a justice system where an accused is guilty until proven innocent vs. innocent until proven guilty” (11).

## The Cultural Shift Toward Zoocentric Virtue Ethics

Accompanying this recognition of animals' intrinsic worth is a virtue ethic based neither on maximizing “the good” (i.e., a utilitarian ethic) nor an obligation to some duty (i.e., a deontological ethic). Instead, “virtue ethics focus on the character traits, or virtues, manifested in proper conduct… includ[ing] respect, humility, generosity, integrity, patience, and, of course, compassion” (12).

Even a cursory review of current events reveals evidence of this zoocentric virtue ethic. As we draft this essay, for example, *The New York Times* is reporting that Tahlequah, a 20-year-old female orca “has been swimming with her daughter's body through choppy seas… on what social media observers and orca researchers call a ‘tour of griefș ” (13) that continued for at least 17 days (14). It's difficult to imagine the “tour” receiving such attention had it not been for the 2013 documentary *Blackfish*, which prompted SeaWorld, 3 years later, to halt its captive breeding program and agree to phase out orca performances in its parks by 2020 (15, 16).

Additional evidence of the powerful cultural shift toward a zoocentric virtue ethic is seen in the growing legal fight over “personhood” for certain animals (5), with perhaps the most noteworthy cases to date involving primates (17). Our increasing recognition of, and concern for, the intrinsic value of animals is also reflected in our expectations for wildlife management, which has traditionally reflected an ecocentric ethic in which the well-being of the collective (e.g., populations, species and ecosystems), rather than any individual of the collective, is the primary goal (11). In 1992, for example, Schmidt (18) proposed a “new philosophical paradigm in wildlife damage management” focusing “on a professional responsibility to *individual* animals in a population, not just ‘abstractș populations or species” (emphasis added). The success of this paradigm shift can also be seen in the compassionate conservation movement's guiding principle of “first do no harm” and “desire to eliminate unnecessary suffering and to prioritize animals as individuals, not just as species” (19).

In 2008, the year Robertson's review was published, a Gallup poll of U.S. adults found that 25% agreed with the statement, “Animals deserve the exact same rights as people to be free from harm and exploitation” (20). This result was unchanged since the previous 2003 poll; in 2015, however, agreement with the statement rose to 32% (20), a 28% increase over the previous result. And a 2011 survey of U.S. pet owners found that 71% of respondents agreed with the statement “Animal shelters should only be allowed to euthanize animals when they are too sick to be treated or too aggressive to be adopted,” while only 25% agreed with the statement “Sometimes animal shelters should be allowed to euthanize animals as a necessary way of controlling the population of animals” (21). When the same statements were presented to respondents of a 2017 national survey that included pet owners—and non-pet owners—agreement with the first statement dropped to 57%, most likely because, unlike in the 2011 survey, an explicit “don't know” option was offered, and selected by 17% of respondents. Agreement with the second statement, however, remained largely unchanged (26%) (22).

It's not surprising that our interest in the humane treatment of companion animals extends beyond the 94.2 million cats with whom 47.1 million Americans share their homes (23) to the millions of community cats with whom we share our neighborhoods. After all, “our moral obligations are clearer to close relations than to those who are further away from us… the wild feral cat is not just another feral animal but the close relative of the animal asleep on people's sofas” (24).

Indeed, evidence of such moral obligations is found in the results of a 2007 Harris Interactive poll commissioned by Alley Cat Allies, in which 81% of U.S. respondents indicated that leaving a community cat alone would “be the more humane option for the cat,” compared to 14% who would opt to have the cat impounded and “put down.” Even when presented with the possibility that the cat “would die in 2 years because it would be hit by a car,” 72% expressed support for leaving the cat alone, 21% for lethal impoundment, with the remaining 7% refusing to answer or indicating that they didn't know (25). In 2014, Beall Research included the same two questions in a more extensive national survey. Seventy-three percent of respondents to the first question expressed a preference for leaving the cat alone, while 9% indicated a preference for lethal impoundment, and 18% refusing to answer or indicating that they didn't know; responses to the follow-up question were 54, 17, and 29%, respectively (26). As these surveys demonstrate, killing a healthy animal out of fear of some *possible* future event, as is sometimes advocated to oppose TNR (27), is out of step with public opinion^1^.

This low level of public support for killing animals as a means of population control (in our animal shelters or our communities) is further evidence of a shift toward a zoocentric virtue ethic that recognizes the intrinsic value of animals beyond any instrumental value to humans, and the considerable role that compassion and empathy play in our “animal control” preferences. This last point is worth highlighting since critics of TNR routinely dismiss its support by animal welfare organizations and the general public as an emotional, but ultimately misguided, response (34–37). As Rawles (10) points out, such accusations are ironic given the rational nature of “the arguments that animal welfarists draw on” from the ethics literature, which “explicitly *disavow* any appeal to emotion, utilizing instead a very hard-nosed appeal to consistency and logical reasoning.”

“In my view, this approach is if anything *too* rational, leaving no room for the legitimate role of emotions in ethical deliberation and underpinned by a mistaken view of what emotions are like” (10, emphasis in original).

## The No-kill Movement Comes of Age

Historically, the management of companion animals was driven largely by the same anthropocentric utilitarian ethical framework used by wildlife managers. As a result, lethal methods were used almost exclusively. As the animal rights movement of the 1970s and 1980s began to focus attention on the intrinsic value of all animals and their right to be treated with compassion (6, 7), the U.S. animal welfare community began calling for the fundamental reform of animal sheltering: “Euthanasia might be a relatively painless end to this journey of terror,” reads one seminal essay, “but each death represents an abject failure—not an act of mercy” (38).

In 2007, a year before Robertson's review was published, Winograd (39) formalized the tenets of “the no-kill movement,” arguing that it “has the potential to end, once and for all, the century-old notion that the best we can do for homeless dogs and cats is to adopt out a few, and kill the rest.” Since then, U.S. cities and states have adopted no-kill resolutions, making public their commitment to saving the animals entering their shelters (40–43). Accompanying such commitments is the recognition that TNR and a suite of related programs (e.g., “working cat” programs, kitten nurseries) are indispensable for achieving no-kill objectives (41, 44). Indeed, the first of the “mandatory programs and services” included in Winograd's “No-Kill Blueprint for Shelters” is TNR.

“For feral cats, TNR is the sole alternative to the mass killing perpetrated in U.S. animal shelters… In fact, because of their unsocial disposition, they are not considered adoption candidates. As a result, there is no other animal entering whose prospects are so grim and outcome so certain. Without TNR, all feral cats who enter shelters are killed” (39).

The protections offered by these programs reflect our evolving ethics; the once-dominant anthropocentric utilitarian framework is being challenged by our recognition of the intrinsic value of cats (owned and unowned alike) and the legitimacy of compassion in shaping our moral obligation to them.

## Support for TNR

Although TNR is controversial (3, 45, 46), even some of its harshest critics concede, “there is little question that cat advocates are winning the war in the court of public opinion” (3). Indeed, the results of public opinion surveys concerning preferred methods of community cat management show strong support for TNR, and for the non-lethal management of community cats more generally. A national survey commissioned by Best Friends Animal Society and conducted by Luntz Global in 2014 found that 68% of respondents preferred TNR, compared to 24% who chose impoundment “followed by lethal injection for any cats not adopted” and 8% who chose “do nothing” (47). Three years later, another national survey asked a nearly identical question with nearly identical results: 72% of respondents chose TNR, compared to 18% who chose impoundment/lethal injection and 11% who chose “do nothing” (22, 48). Similar levels of support have been observed at the state (49) and local levels (50).

Other surveys on the subject indicate lower levels of support for TNR; however, these apparent discrepancies are easily understood when the survey designs are scrutinized. Ash and Adams (51), for example, found that 55% of Texas A&M University employees preferred TNR to manage cats on campus. However, the “removal” option chosen by 42% of respondents was actually two options: “either humanely put to sleep or adopted out to a home” (52), with no way to parse the results. Similarly, residents of Athens-Clarke County, Georgia, were asked to rate the acceptability of four options (including “educate the public about feral cats and wildlife”), rather than select one preferred management method (or rank multiple options). As a result, the observation that “cat sanctuaries were found to be the most acceptable option to reduce feral cat populations (56%), followed by TNR (49%) and capturing and euthanizing cats (44%)” (53) tells us little about management *preferences*. On the other hand, it's clear once again—from both surveys—that there's little public support for lethal management methods.

A survey of the general public in four Florida counties found that 54% of respondents preferred TNR, compared to 25% who preferred placement in a long-term no-kill shelter and 15% preferring to trap and “euthanize” cats (54). In fact, the “long-term no-kill shelter” option is, like the sanctuary option referred to above, largely a false choice;^2^ shelters committed to reducing feline intake and killing rarely house cats long-term and are instead turning to shelter-based TNR, often called return-to-field programs (30, 54). Regardless, 85% of the “general public” (including the presumed 6% who chose “leave alone”) preferred the non-lethal options offered.

Other surveys investigating public support for TNR have reported lethal methods to be more popular than non-lethal methods. Loyd and Miller (55), for example, found that 52% of Illinois homeowners “preferred capture and euthanasia for feral cat management, 27% capture-neuter-return, 18% capture and keep in shelter, and 3% chose ‘other.ș ” However, a review of the original survey upon which these results are based (56), and its subsequent analysis, reveals a survey sample that fails to accurately represent Illinois homeowners. Chicago area residents (37% supported TNR, 38% supported “capture and euthanize,” and 20% supported “capture and retain in shelter”) were underrepresented by nearly 50% compared to other Illinois residents. And hunters, who were found to be less supportive of TNR (13% TNR, 73% lethal, 12% shelter), were overrepresented by a factor of almost 10. Similar sampling issues undermine the claim by Lohr and Lepczyk (57) that “live capture and lethal injection was the most preferred technique and trap-neuter-release was the least preferred technique for managing feral cats” in Hawaii. In fact, 82.5% of the study's “random residents” sample “lived in a rural area or small town” whereas “only 10% of Hawaii's population live in rural areas with fewer than 50,000 residents” (58). Moreover, 24% of “random residents” indicated that they hunted at least once annually, more than 34 times the expected rate (0.7%) based on hunting licenses purchased in 2009 (58). Thus, these surveys tell us very little about the general public's preference for managing community cats.

Support for TNR extends beyond the general public, too. The American Public Health Association's Veterinary Public Health Special Primary Interest Group, for example, “support[s] well-designed [TNVR^3^] programs as the preferred method of management wherever feasible” (59). And the National Animal Care & Control Association “recognizes that in some circumstances, alternative management programs, including [TNVR] programs may be effective, and recommends that each agency assess the individual need with their community and respond accordingly” (60).

In 2016, the American Veterinary Medical Association (AVMA) shifted its official position on the issue in a direction more favorable to TNR. Although the organization notes that “there is currently not consensus around what an ultimate solution will look like,” AVMA now “encourages the use of non-lethal strategies as the initial focus for control of free roaming abandoned and feral cat populations. Public, private, and not-for-profit humane organizations and individuals must make every effort to promote adoption of acceptable unowned cats and implement sterilization programs.” AVMA's previous position statement, published in 2012, made no mention of non-lethal methods and “neither endorse[d] nor oppose[d] appropriately managed cat colony programs” (61). And more recently, the American Bar Association approved a resolution “support[ing] the adoption of laws and policies supportive of TNVR programs with the intent of decreasing community cat populations and improving public health and safety…” (62).

Such endorsements reflect the considerable and varied memberships of the individual organizations—and by extension, the public they serve. Again, such clear support for TNR reflects the growing consensus that community cats have intrinsic value and deserve to be treated with compassion.

## Conclusions

The momentum we're witnessing in the no-kill movement generally, and TNR in particular, reflect a profound shift away from an anthropocentric utilitarian ethical framework toward a zoocentric virtue-based ethical framework that recognizes the intrinsic value of animals beyond any instrumental value to humans and our moral obligation to treat them with compassion. Ten years ago, Robertson (1) highlighted the need for additional scientific research to “improv[e] current control methods” and called for both TNR programing and education to reduce community cat numbers. As this volume—and the works cited herein—demonstrate, the TNR literature has greatly expanded over the past 10 years; and programing, education, and outreach efforts continue to expand as TNR is adopted across the U.S., in communities large and small, urban and rural.

TNR's momentum and broad public support suggest almost an arc-of-history inevitability, and brings to mind a quote from Vucetich et al. (11): “Although the principles of social justice were developed with humans in mind, social justice's roots in intrinsic value suggests that it might be expanded and adapted to better understand what constitutes appropriate relationships between humans and the rest of the natural world.”

## Author Contributions

Both authors contributed equally to the overall development of this essay. PW contributed the majority of content related to public opinion surveys while JS contributed the majority of content related to various ethical philosophies.

### Conflict of Interest Statement

In recognition of Frontiers' policy and our ethical obligations as authors, we acknowledge that one of the authors PW is employed by Best Friends Animal Society, advocating for the protection of domestic cats via public policy initiatives. The remaining author declares that the research was conducted in the absence of any commercial or financial relationships that could be construed as a potential conflict of interest.

## Footnotes

^1^This “better-off-dead” philosophy, as it's sometimes called, is not supported by the growing body of evidence demonstrating that the vast majority of community cats are healthy (28–33) and is inconsistent with a zoocentric virtue ethic that recognizes and respects the intrinsic worth of individual animals.

^2^TNR critics Marra and Santella (3) significantly understate the case when they acknowledge that “sanctuaries do not appear to be a model that can be scaled to meet the current need.”

^3^APHA, NACA, and ABA use the term *TNVR* to make explicit the vaccination component of TNR. Although vaccination against rabies is common practice for TNR programs located in areas of the U.S. where rabies in cats occurs most frequently—and is considered best practice for *all* TNR programs—it is not yet universal. Nor is the term *TNVR*; we've therefore chosen to use the more common term *TNR* throughout this paper.
